# Renal cold storage followed by transplantation impairs expression of key mitochondrial fission and fusion proteins

**DOI:** 10.1371/journal.pone.0185542

**Published:** 2017-10-04

**Authors:** Nirmala Parajuli, Stephen Shrum, Julia Tobacyk, Alex Harb, John M. Arthur, Lee Ann MacMillan-Crow

**Affiliations:** 1 Department of Pharmacology and Toxicology, University of Arkansas for Medical Sciences, Little Rock, AR, United States of America; 2 Department of Internal Medicine, University of Arkansas for Medical Sciences, Little Rock, AR, United States of America; University of Toledo, UNITED STATES

## Abstract

**Background:**

The majority of transplanted kidneys are procured from deceased donors which all require exposure to cold storage (CS) for successful transplantation. Unfortunately, this CS leads to renal and mitochondrial damage but, specific mitochondrial targets affected by CS remain largely unknown. The goal of this study is to determine whether pathways involved with mitochondrial fusion or fission, are disrupted during renal CS.

**Methods:**

Male Lewis rat kidneys were exposed to cold storage (CS) alone or cold storage combined with transplantation (CS/Tx). To compare effects induced by CS, kidney transplantation without CS exposure (autotransplantation; ATx) was also used. Mitochondrial function was assessed using high resolution respirometry. Expression of mitochondrial fusion and fission proteins were monitored using Western blot analysis.

**Results:**

CS alone (no Tx) reduced respiratory complex I and II activities along with reduced expression of the primary mitochondrial fission protein, dynamin related protein (DRP1), induced loss of the long form of Optic Atrophy Protein (OPA1), and altered the mitochondrial protease, OMA1, which regulates OPA1 processing. CS followed by Tx (CS/Tx) reduced complex I, II, and III activities, and induced a profound loss of the long and short forms of OPA1, mitofusin 1 (MFN1), and mitofusin 2 (MFN2) which all control mitochondrial fusion. In addition, expression of DRP1, along with its primary receptor protein, mitochondrial fission factor (MFF), were also reduced after CS/Tx. Interestingly, CS/Tx lead to aberrant higher molecular weight OMA1 aggregate expression.

**Conclusions:**

Our results suggest that CS appears to involve activation of the OMA1, which could be a key player in proteolysis of the fusion and fission protein machinery following transplantation. These findings raise the possibility that impaired mitochondrial fission and fusion may be unrecognized contributors to CS induced mitochondrial injury and compromised renal graft function after transplantation.

## Introduction

Renal transplantation has been a clinical reality for almost 50 years, and improved success in recent years is attributed to the advent of tissue-typing protocols and superior immunosuppressive regimens. While immune mechanisms are clearly important to allograft survival, the existence of alloantigen independent mechanisms is undeniable. Kidneys from living donors have better long-term graft outcome [1–3] than those from deceased donors, regardless of tissue match. The key variable is cold storage (CS); kidneys from living donors are generally excluded from CS, whereas those from deceased donors are subjected to cold preservation to allow transportation to recipient, and time for tissue typing and cross-matching procedures prior to transplantation. Tragically, last year ~34% of harvested donor kidneys were discarded or not transplanted (UNOS website data) in part due to CS-mediated injury.

The purpose of CS is to lower the kidney’s metabolic activity and oxygen demand during the wait for transplantation, but nevertheless, injury occurs (for review, please see [4]). Several studies show that the longer a donor kidney is exposed to CS, the poorer the transplant outcome [5–8]. The inverse correlation between duration of CS and graft survival offers compelling support for the CS injury premise. The adverse effects of renal CS are evident in animal models [9–12] and cellular models [13–16]. For example, rat kidneys exposed to CS show extensive injury to cortical and medullar compartments, and marked mitochondrial damage. Cells exposed to CS sustain mitochondrial injury and cell death [12;13;15;17]. However, the mechanisms that mediate renal CS-induced mitochondrial damage remain elusive and are the focus of this study.

Several studies, including our own, have revealed that renal CS injury induces reactive oxygen species (ROS) and this contributes to impaired mitochondrial respiration, loss of ATP, and cell death [11;13;14;18–23]. But how this occurs, and identification of specific mitochondrial target proteins/pathways altered following transplant, remain elusive. Mitochondria are dynamic organelles, which continually undergo morphological changes, including fission and fusion, and mitophagy, ultimately leading to biosynthesis of new functional mitochondria (biogenesis) [24–28]. It is clear that an imbalance of mitochondrial fission and fusion is observed in many disease states, and disruption of fusion limits the response to acute toxic stress by preventing efficient respiration and compensatory mixing of functional mitochondria with damaged ones. Mitochondrial fission and fusion are regulated by a group of GTPase proteins. DRP1 (dynamin-related protein 1) promotes fission by interacting with receptor proteins in the mitochondrial outer membrane. Proteins called mitofusins (MFN1/2), and optic atrophy protein type 1 (OPA1) are GTPase’s that participate in the fusion process of the outer and inner membranes. OPA1 exists in long and short forms, which are regulated by two inner membrane proteases (OMA1 and YME1L) [29]. The long form of OPA1 (L-OPA1) is required for efficient fusion, whereas accumulation of the short form (S-OPA1) can promote fission. Stressful conditions, often associated with mitochondrial membrane depolarization, lead to activation of OMA1 (an ATP independent protease with activities overlapping with the m-AAA protease) causing proteolysis of the long to short form of OPA1 resulting in impaired fusion and mitochondrial fragmentation [30–32]. YME1L (an ATP dependent i-AAA protease) is thought to be constitutively active towards OPA1 cleavage, but can be degraded by OMA1 during stress [31;33].

Abnormalities of mitochondrial fission or fusion during renal CS/Tx have not been reported previously. We have data suggesting, for the first time, the relationship between extensive mitochondrial dysfunction and decreased OPA1, MFN1/2, DRP1, MFF, YME1L and an altered conformation of OMA1 in kidneys exposed to CS/Tx compared to transplantation without CS. These findings raise the possibility that impaired mitochondrial fission/fusion may be an unrecognized contributor to CS-induced injury and compromised renal graft function after transplantation.

## Methods

### Ethical statement and animals

Male Lewis rats weighing 200–250g were used as transplant donors and recipients in this study. All animal protocols were approved by the Institutional Animal Care and Use Committee (IACUC; protocol #3617), at the University of Arkansas for Medical Sciences (UAMS), and all animal experiments described below were performed in compliance with the IACUC at UAMS using NIH guidelines (please refer to S1 File containing ARRIVE checklist). Rats were housed 3 per cage before surgery, after surgery they were placed in individual cages in a climate-controlled room with 12-hr artificial light/dark cycle. Rats were euthanized by exsanguination via withdrawal of blood from the posterior vena cava followed by ligation of the aorta while under isoflurane anesthesia (5% induction and 2% during procedure or surgery). Based on a power analysis of our preliminary CS/Tx data, an “n” of 10 animals is needed for each group to detect a 20% change in mitochondrial function (from shams) with a power = 0.80, α = 0.05 and β = 0.20. Based on power analysis of our published and preliminary data for the autotransplant model an “n” of 8 is needed to detect a 20% change in serum creatinine with a power = 0.80, α = 0.05 and β = 0.20. The research team used an unbiased approach of blinded analysis when possible.

### Syngeneic rat renal transplant model with cold storage

**Fig 1:**
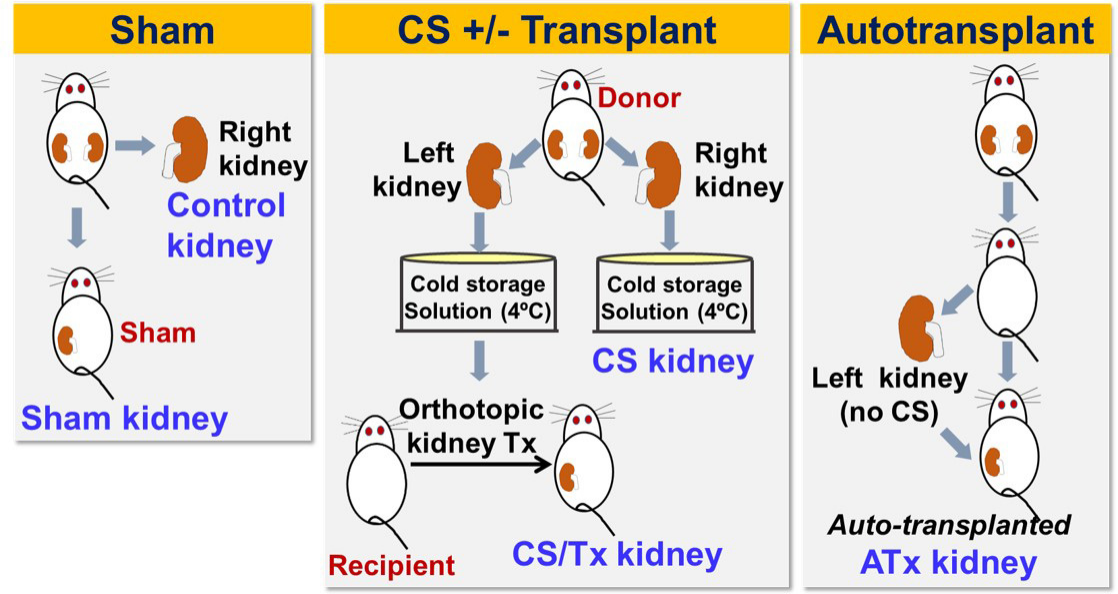
Schematic of five rat surgical groups used in this study. The left panel depicts the sham surgery, in which the right kidney was removed (**control kidney**) and the rat survives with only the left native kidney (**sham kidney**). The middle panel shows transplant surgery using donor kidneys (left and right), which were harvested and exposed to cold storage solution for 18 hrs. The right kidney was saved as CS control (**CS kidney**) and the left kidney was transplanted in a new recipient rat, in which both native kidneys were removed so that the kidney function depends on the transplanted donor kidney (**CS/Tx kidney**). The right panel shows autotransplant surgery, in which both native kidneys were removed in a rat, but the left native kidney was transplanted immediately back to the same rat. This kidney was saved as autotransplanted kidney (**ATx kidney**) and served as a control transplant kidney without CS for the CS/Tx kidney.

Orthotopic renal transplant surgery was performed in male Lewis rats as illustrated in **Fig 1**and in our recent publication [23]. For the donor rat surgery, rats were anesthetized using isoflurane, and the left and right kidneys were flushed and stored in University of Wisconsin (UW) solution at 4^o^ C for 18 hr. The right kidneys of donor rats are referred to as **CS group** (n = 10). For the recipient rat surgery, rats were anesthetized using isoflurane, the native left kidney was removed, and the donor left kidney (exposed to CS) was transplanted using end-to-end anastomosis as described previously [23]. The surgical ischemia time was less than 45 min. The right native kidney was immediately removed, making renal function dependent on the transplanted left kidney. The ureter was anastomosed end-to-end over a 5 mm PE-50 polyethylene stent. Postoperatively, the animals were given 0.9% (w/v) NaCl in the abdominal cavity and placed under a heating lamp to recover from the anesthesia. All efforts were made to minimize suffering, rats were given buprenorphine (0.06 mg/kg, SC) for pain (first dose immediately after the surgery, second dose was given 4 hr after surgery, and the final dose given 16 hr after surgery). Body temperature, pulse and respiratory rate were observed until the animal regained full consciousness, a righting reflex, and ability to stand erect. The rat was then placed in a cage and provided free access to food and water. Rats showing signs of distress were immediately euthanized while under anesthesia. After 24 hr of reperfusion, the transplanted left kidney and blood were collected under anesthesia and saved as **18hr cold storage plus transplantation (CS/Tx)** group (n = 10). The rat survival rate was greater than 95% for the CS/Tx groups. Animals were not randomized other than if a donor or recipient rat kidney displayed multiple renal arteries or veins making the transplant procedure more difficult, these rats were used for control or sham animals described below.

### Control groups (Control, Sham, and Autotransplant)

#### Autotransplant surgery

The autotransplant (ATx) surgery was included in these studies so that the impact of CS could be isolated from the impact of transplant surgery alone with regard to alterations in mitochondrial fusion/fission protein expression. ATx is performed as described in the recipient surgery method, except that the left kidney was removed, flushed with saline and immediately transplanted in the same rat without CS exposure, followed by right nephrectomy (**Fig 1**). After 24 hr, the transplanted kidney was harvested under anesthesia, and the organs were referred to as **Autotransplant (ATx)** group (n = 8).

#### Sham surgery

Rats underwent identical surgery (right nephrectomy), but without the renal transplantation (Sham operation, **Fig 1**). The right kidney from a healthy rat was removed and saved as **Control** kidney (n = 10). The left kidney remained functioning for 24 hr, and then the sham kidney and blood were harvested and saved as the **Sham** group (n = 10).

After harvesting, kidneys were immediately processed for HRR studies, histology (formalin fixed), or flash frozen for biochemistry/western blots. The ‘control’ kidney was compared to the CS alone kidney since both kidneys were harvested from healthy rats. The ‘sham’ kidney served as the control for both transplant models (ATx and CS/Tx) since all underwent a nephrectomy (removal of right kidney).

### Blood chemistry

Blood chemistry was determined in heparinized blood (arterial) using a hand-held clinical chemistry analyzer, iSTAT^TM^, and cartridges (CHEM8^+^) as described by the manufacturer (Vetscan®, Abaxis, USA).

### Transmission electron microscopy

Fresh kidneys were cut into small pieces (~1 mm^3^) representing cortex and fixed with fixative solution (2% glutaraldehyde and 4% paraformaldehyde made in cacodylate buffer (0.1M, pH 7.4)) for 2hr at room temperature followed by storing at 4°C. The fixed biopsies were then sent to the histology core laboratory at Georgia Health Sciences University for further processing and analysis.

### High resolution respirometry (HRR)

Mitochondrial respiratory complex activity was measured in the saponin permeabilized renal biopsies (representing cortex and medulla) by high resolution respirometry (HRR) (Oroboros instruments—Oxygraph-2k, Innsbruck, Austria), according to substrate-inhibitor-titration (SIT) protocol as described earlier [19;34;35]. Briefly, representative renal biopsies (cortex and medulla) of the kidneys were incubated with 100 μg/ml Saponin prepared in MiRO5 buffer, followed by 3x washes with MiRO5 buffer [60 mM K-lactobionate, 0.5 mM EDTA, 3 mM MgCl2, 20 mM Taurine, 10 mM KH2PO4, 20 mM HEPES, 110 mM BSA, and 1 g/L sucrose, pH 7.0]. Mitochondrial respiration was initiated by adding 2 mM malate and 10 mM glutamate and maximum active respiration was achieved by adding 2.5 mM ADP. Rotenone (0.1 mM) was then added to completely inhibit complex I respiration. To measure complex II and III respiration 10 mM succinate was added followed by 2mM malonate to inhibit complex II respiration (complex II activity), and 10 μM antimycin A to inhibit complex III respiration (complex III activity). Inhibitor concentrations were selected based on experimental determination of doses needed to maximally reduce substrate-induced respiration. Finally, data analysis was done using DATLAB 4.2 software (Oroboros), and tissue respiration was shown as oxygen flux (pmol/mg/s).

### Renal extract preparation for Western blot analysis

Renal extracts from whole kidney homogenates were prepared by using radioimmuno-precipitation assay (RIPA) buffer (Pierce, USA) with 1 mM PMSF (Sigma, USA), 1.2 mM Na_3_VO_4_ (Sigma, USA), 2.5 mM NaF (Sigma, USA), 1mM DTT (Sigma, USA) and protease inhibitor cocktail (Pierce, USA). Protein concentrations were determined by Coomassie Plus Protein Assay Reagent (Pierce, USA). Renal extracts (30 **μ**g) were resolved onto SDS-PAGE gel and then transferred to PVDF membrane. Western blot analysis was performed using antibodies against proteins: DRP1 (1:1000, BD Bioscience, #611112); MFF (1:1000, Abcam, # ab81127); MFN1 (1:1000, Abcam, #ab57602); MFN2 (1:1000, Abcam, # ab124773); OPA1 (1:1000, Abcam, #ab42364); YME1L (1:1000, Abgent, #AP4882a); OMA1 (1:1000, Santa Cruz, # sc-515788); Core 2 (1:1000, Abcam, #ab14745); MnSOD (1:1000, Millipore, #06–984) and β-actin (1:1000, Sigma, # A5441). Actin expression was used as a loading control for all western blot experiments. Probed membranes were washed three times and immune-reactive proteins were detected using horseradish peroxidase conjugated secondary antibodies (Seracare KPL, USA) and enhanced chemiluminescence (Thermoscientific, USA). Densitometry evaluation on scanned membrane was performed using AlphaEase FC software.

### Statistical analysis

Results are presented as mean ± standard error of the mean (S.E.M.) using Graph Pad Prism software. Mitochondrial respiration data and western densitometry analysis between all transplant groups (Sham, ATx, CS/Tx) were analyzed by a one-way factorial analysis of multivariance (ANOVA) followed by Tukey’s post-hoc test using multiple group comparisons. For renal function analysis an unpaired Student's *t*-test was used when comparing differences between the mean of two groups (Control versus CS alone, and Sham vs. CS/Tx) at a 95% level of confidence. Likewise, western densitometry analysis comparing Control versus CS alone was done using an unpaired Student’s *t*-test. Differences with a *P* value < 0.05 were considered statistically significant.

## Results

### Cold storage exacerbates mitochondrial and renal damage after transplantation

**Fig 2:**
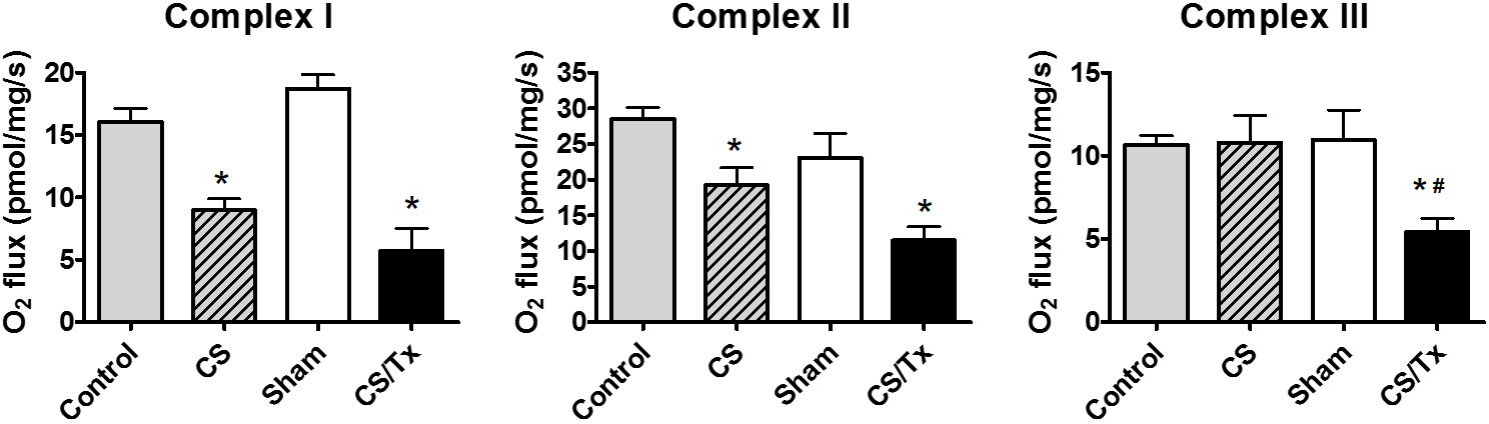
Cold storage alone and combined with transplantation reduces mitochondrial function. Graph showing respiratory complex I, II, and III activity of the electron transport chain using HRR in kidney biopsies from control, sham, CS alone, and CS combined with transplantation (CS/Tx). Values were expressed as Mean ± S.E.M. (n = 5). Difference between the mean of the groups were compared using a one-way factorial analysis of multivariance (ANOVA) followed by Tukey’s post-hoc test for multiple group comparisons; *, indicates means are significantly different (P < 0.05), when compared between control and CS alone or between Sham and CS/Tx. Similarly, #, indicates means are significantly different (P < 0.05), when compared between CS alone and CS/Tx.

**Fig 3:**
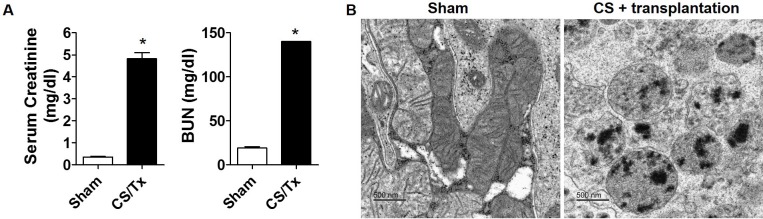
Cold storage plus transplantation impairs renal function and mitochondrial morphology. **(A)** Serum creatinine and blood urea nitrogen (BUN) from the sham and 18 hr CS/Tx rats were analyzed using hand-held clinical chemistry analyzer (iSTAT^TM^) and Chem8^+^ cassettes as described in materials and methods. Values were expressed as Mean ± S.E.M. (n = 4). The unpaired Student’s t test was used to compare the means between sham and CS/Tx; * indicates means are significantly different from sham (P < 0.05). **(B)** Electron micrographs revealed normal mitochondria in sham kidneys (cortical region), but 18 hr CS/Tx kidneys show rounded, fragmented mitochondria with dense aggregates. Images are representative of n = 3 in each group; bar, 500 nm. TEM was performed at the Georgia Reagents TEM Core Facility.

The activities of renal mitochondrial respiratory complexes I, II and III were assessed by high resolution respirometry (HRR), which has the advantage of using an intact tissue biopsy, rather than relying on mitochondrial isolation. Isolated rat kidneys were exposed to CS alone (18 hr) which resulted in significant inhibition of complexes I and II activity when compared to controls (rats with both native kidneys intact) (**Fig 2**). Rat kidneys exposed to CS (18 hr) followed by transplantation in a new recipient rat (CS/Tx) resulted in a greater inactivation of complex III when compared to sham kidneys (rats with removal of the right kidney, but no CS or Tx) or CS alone kidneys. Compared to kidneys from sham rats, kidneys subjected to CS/Tx showed impaired renal function 24 hr after transplantation with increased serum creatinine and BUN levels (**Fig 3A**). Our earlier study showed that ATx did not alter mitochondrial complex activity, but induced renal injury when compared to sham [23]. The ultrastructure of mitochondria was assessed using transmission electron microscopy (Georgia Reagents TEM Core Facility). Representative images of sham kidneys show normal mitochondrial structure (**Fig 3B**). However, CS/Tx resulted in profound fragmentation and rounding of renal mitochondria (**Fig 3B**).

### Cold storage alone disrupts renal mitochondrial fission proteins

**Fig 4:**
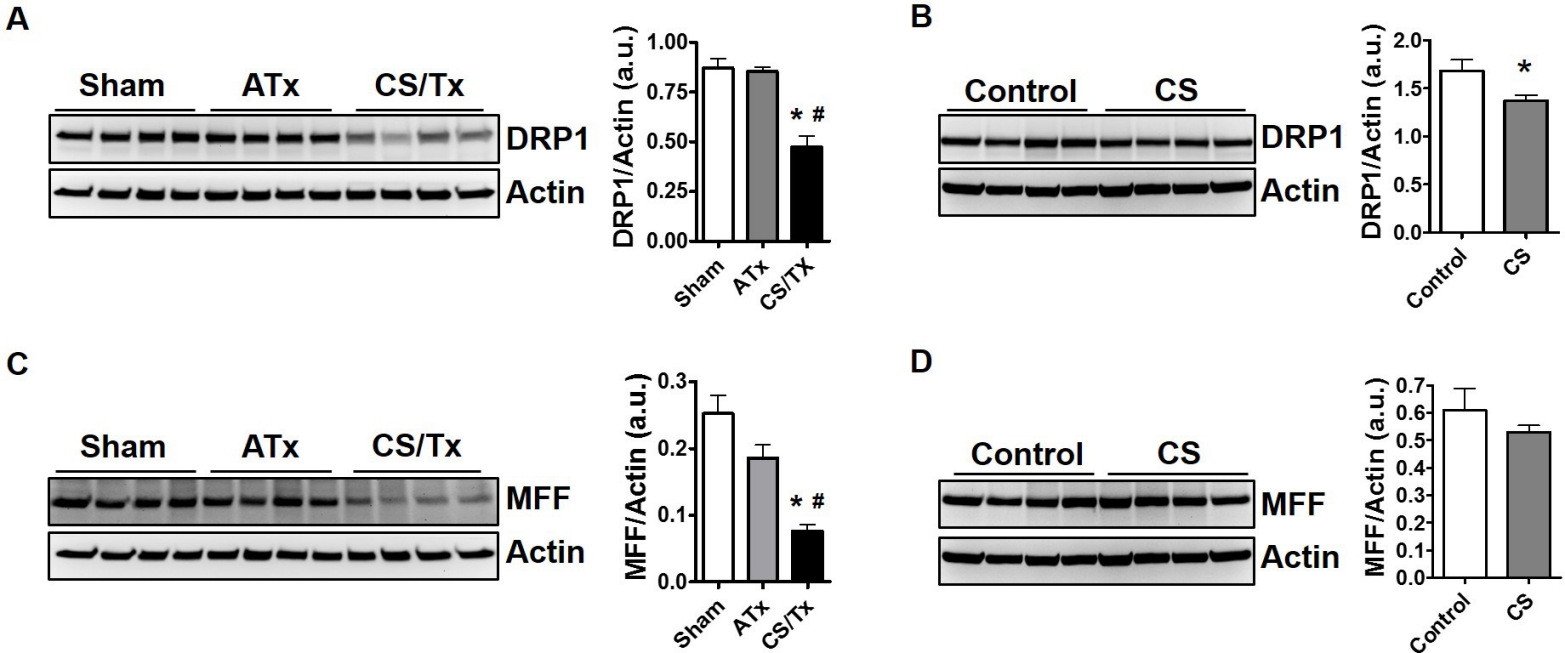
Cold storage alone and cold storage plus transplantation alters mitochondrial fission proteins, but autotransplantation has no effect. Renal extracts (30 ug) were resolved on SDS-PAGE gels and immunoblotted. Representative DRP1 western blot showing distinct protein bands of DRP1 (~75 kDa) in Sham, ATx, and CS/Tx kidneys **(A)** as well as control and CS alone kidneys **(B).** Actin was used as a loading control. Densitometry evaluation of each blot (normalized to actin) is shown on the right panel. Representative MFF western blot showing distinct protein bands of MFF (~38 kDa) in Sham, ATx, and CS/Tx kidneys CS/Tx **(C)** as well as control and CS alone kidneys **(D).** Actin was used as a loading control. Densitometry evaluation of each blot (normalized to actin) is shown on the right panel. Values were expressed as Mean ± S.E.M. (n = 4). Unpaired Student’s t test was used to compare the means between control and CS kidneys. One-way ANOVA followed by Tukey’s post-hoc test for multiple group comparisons was used to compare the means between sham, ATx, and CS/Tx kidneys; * indicates means are significantly different (P < 0.05) when compared to control or sham and # indicates means are significantly different (P < 0.05) when compared to ATx.

In an attempt to dissect pathways responsible for the profound mitochondrial fragmentation during CS/Tx, rat tissues from the experiment described in Fig 2 were used to assess protein expression of DRP1, the primary protein within the mitochondrial fission cascade, as well as the primary DRP1 receptor protein, called mitochondrial fission factor (MFF). Results are presented by comparing sham kidneys (rats with one kidney-post nephrectomy) compared to ATx or CS/Tx since all rat groups have one kidney. Similarly, kidneys exposed to CS alone were compared to control rat kidneys. Autotransplantation (ATx) had no effect on DRP1 expression (~75 kDa), but CS combined with Tx (CS/Tx) significantly reduced DRP1 expression compared to sham kidneys (**Fig 4A**). Interestingly, CS alone also significantly reduced DRP1 expression (~75 kDa) compared to control kidneys (**Fig 4B**). MFF expression (~38 kDa) was only reduced with combined CS/Tx (**Fig 4C**), not after CS alone or ATx (**Fig 4C & 4D**). Actin expression was used as a loading control for all western blot experiments.

### Cold storage alone disrupts renal mitochondrial fusion proteins

**Fig 5:**
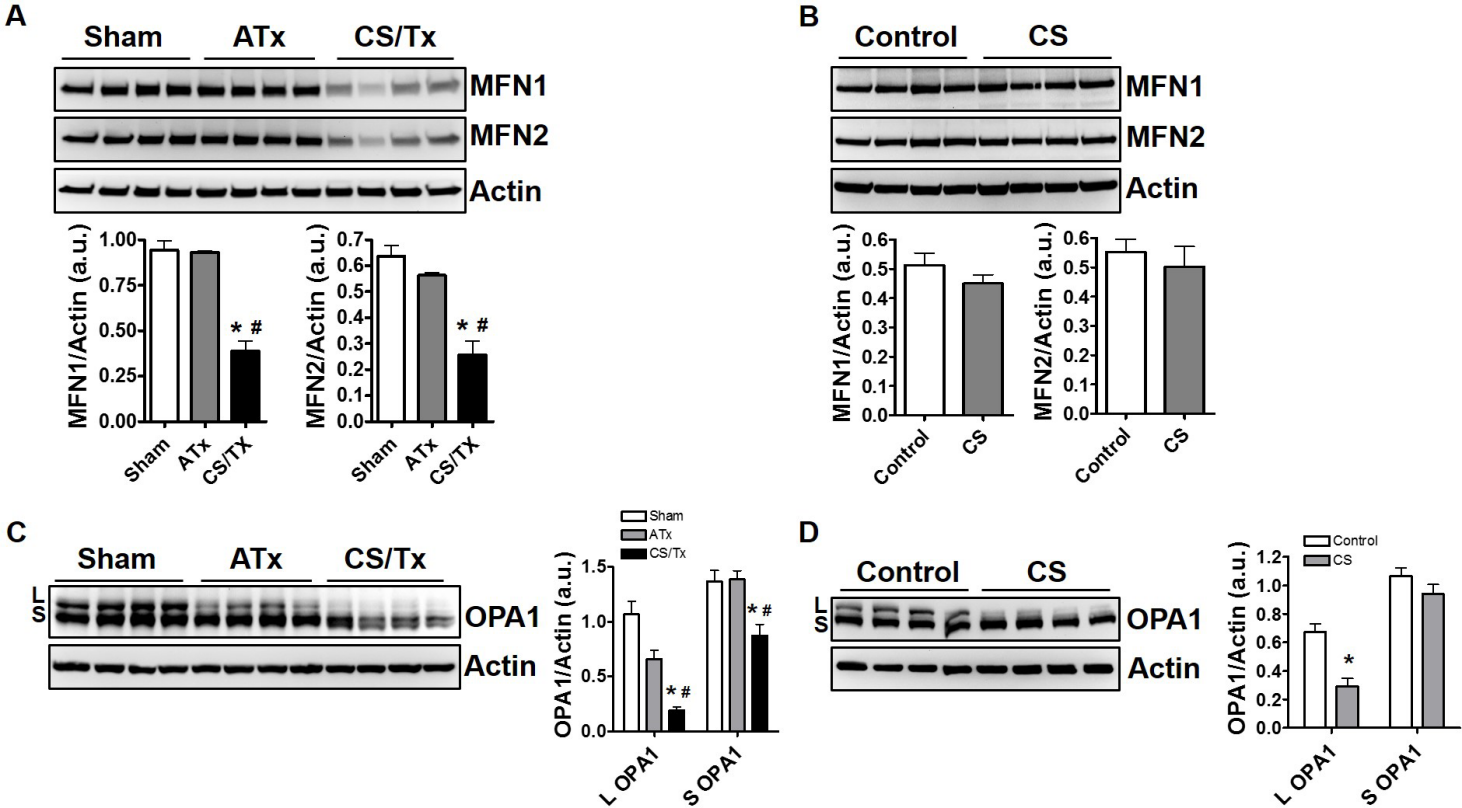
Impact of cold storage alone, combined cold storage plus transplantation, and autotransplantation on mitochondrial fusion protein expression. Renal extracts (30 ug) were resolved on SDS-PAGE gels and immunoblotted. Representative MFN 1 and MFN2 western blots showing distinct protein bands of both MFN1/2 (~75 kDa) in Sham, ATx, and CS/Tx kidneys **(A)** as well as control and CS alone kidneys **(B).** Actin was used as a loading control. Densitometry evaluation of each blot (normalized to actin) is shown on the right panel. Representative OPA1 western blot showing long form of OPA1 (L, ~95 kDa) and short form of OPA1 (S, ~75 kDa) in Sham, ATx, and CS/Tx kidneys CS/Tx **(C)** as well as control and CS alone kidneys **(D).** Actin was used as a loading control. Densitometry evaluation of each blot (normalized to actin) is shown on the right panel. Values were expressed as Mean ± S.E.M. (n = 4). Unpaired Student’s t test was used to compare the means between control and CS kidneys. One-way ANOVA followed by Tukey’s post-hoc test for multiple group comparisons was used to compare the means between sham, ATx, and CS/Tx kidneys; * indicates means are significantly different (P < 0.05) when compared to control or sham and # indicates means are significantly different (P < 0.05) when compared to ATx.

We next evaluated key proteins involved with mitochondrial fusion including mitofusins (MFN1/2) and optic atrophy protein type 1 (OPA1), which are GTPase’s that regulate the fusion process of the outer and inner membranes, respectively. Neither ATx nor CS alone (18 hr) altered expression of outer membrane fusion proteins, MFN1 or MFN2 (both ~75 kDa); however, CS combined with Tx (CS/Tx) significantly lowered expression of both MFNs (**Fig 5A & 5B**). Conversion of the long form of inner mitochondrial fusion protein OPA1 (L; ~95 kDa) to the short form (S; ~75 kDa) is indicative of OPA1 proteolysis and impaired mitochondrial fusion. Interestingly, CS alone induced conversion of the long form of OPA1 (L; ~95 kDa) to the short form (S; ~75 kDa) (**Fig 5D**) suggesting reduced inner membrane fusion during CS. However, CS/Tx lead to extensive loss of both the long and short form of OPA1 (**Fig 5C**) suggesting severe impairment of inner mitochondrial membrane fusion.

### Role of key mitochondrial proteases during renal cold storage

**Fig 6:**
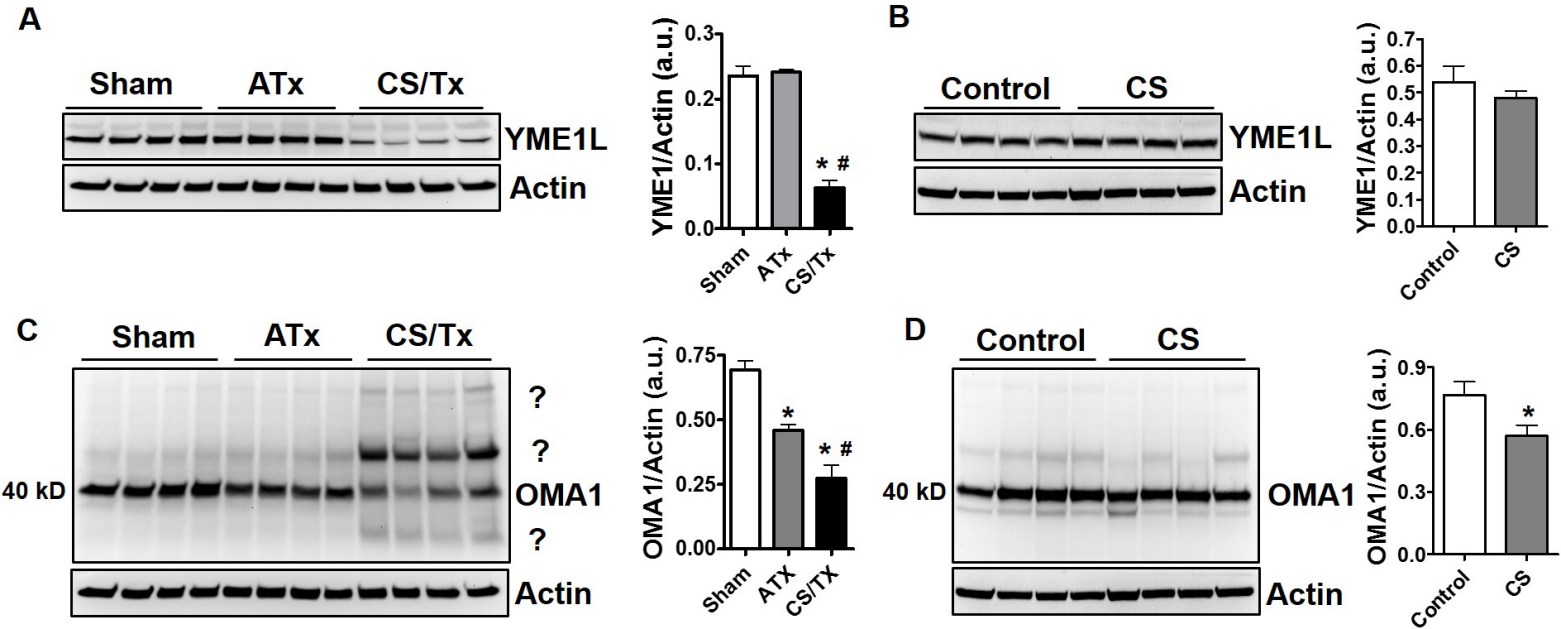
Alteration of OPA1 regulator proteases (YME1L and OMA1) after cold storage plus transplantation. Renal extracts (30 ug) were resolved on SDS-PAGE gels and immunoblotted. Representative YME1L western blot showing distinct protein bands of YME1L (~60 kDa) in Sham, ATx, and CS/Tx kidneys **(A)** and in control and CS alone kidneys **(B).** Actin was used as a loading control. Densitometry evaluation of each blot (normalized to actin) is shown on the right panel. Representative OMA1 western blot showing distinct protein bands of OMA1 (~40 kDa) after CS/Tx **(C)** and CS alone **(D).** Multiple higher and lower molecular weights of OMA1 reactivity (?) were also visible after CS/Tx. Actin was used as a loading control. Densitometry evaluation of 40 kDa OMA1 (normalized to actin) is shown on the right panel. Values were expressed as Mean ± S.E.M. (n = 4). Unpaired Student’s *t* test was used to compare the means between control and CS kidneys. One-way ANOVA followed by Tukey’s post-hoc test for multiple group comparisons was used to compare the means between sham, ATx, and CS/Tx kidneys; * indicates means are significantly different (P < 0.05) when compared to control or sham and # indicates means are significantly different (P < 0.05) when compared to ATx.

As mentioned earlier, OMA1 and YME1L are mitochondrial proteases known to cleave OPA1 to the short form. OPA1 cleavage is commonly used as an indirect assay for activation of these proteases, since there are no sensitive or specific activity assays available. Interestingly, CS/Tx lead to a reduction of YME1L, while CS alone or ATx did not alter YME1L protein levels (**Fig 6A & 6B**). We show decreased OMA1 expression at its predicted molecular weight of 40 kDa after ATx and CS alone, and a more robust decline after CS/Tx (**Fig 6C & 6D**). Decreased OMA1 protein expression is sometimes used to assess OMA1 activity since it is known that OMA1 can cleave itself [31]. Surprisingly, OMA1 also appeared to form higher molecular weight aggregates only after CS/Tx (**Fig 6C ‘?’).** The reason or function of these OMA1 aggregates remains unknown but might be involved with an aberrant activity of OMA1 during CS/Tx.

**Fig 7:**
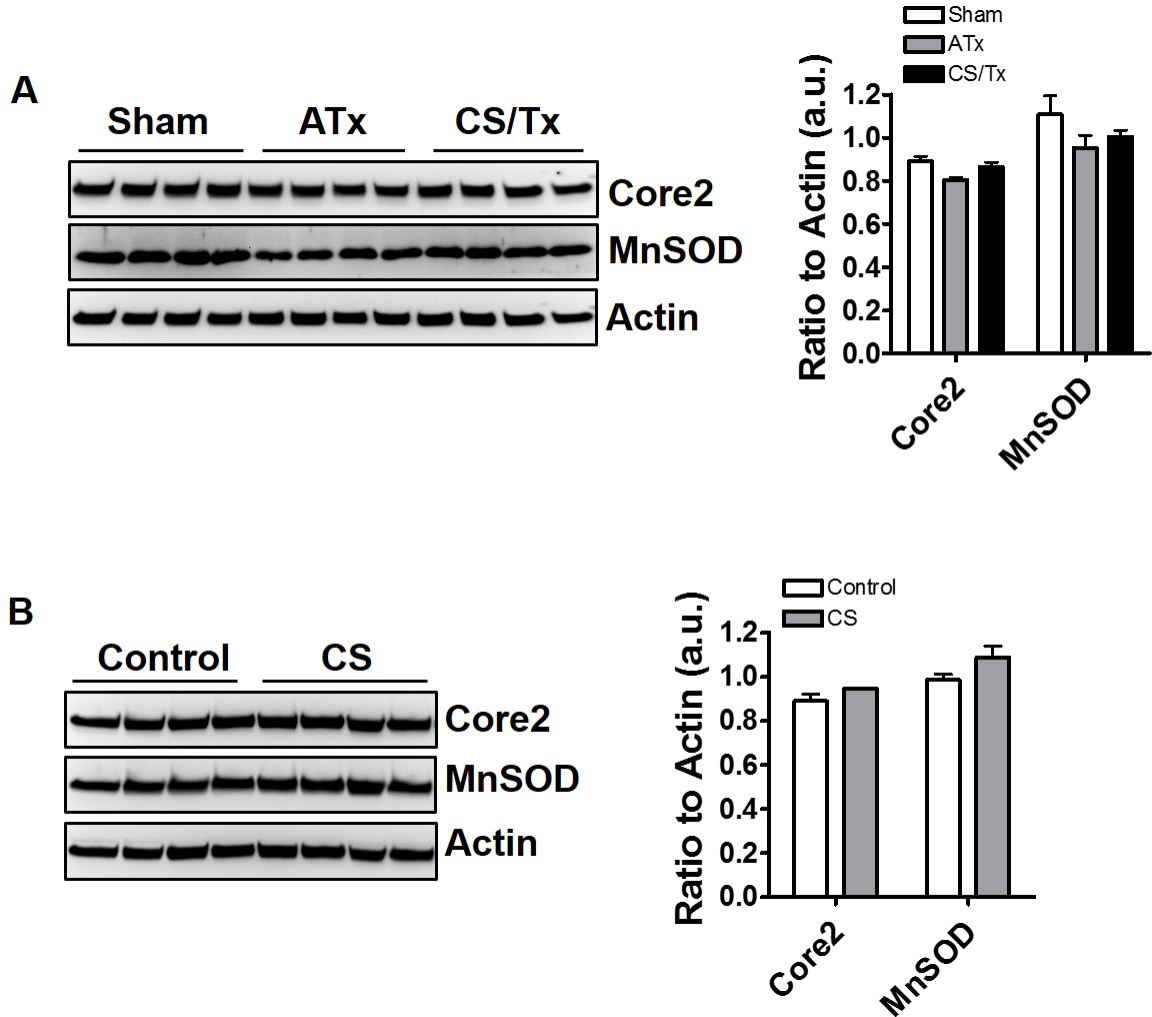
Renal cold storage alone or cold storage plus transplantation does not decrease all mitochondrial protein expression. Renal extracts (30 ug) were resolved on SDS-PAGE gels and immunoblotted. Representative Core2 and MnSOD western blots showing distinct protein bands of Core2 (~49 kDa) and MnSOD (~ 24 kDa) in Sham, ATx, and CS/Tx **(A)** and comparing control to CS alone **(B).** Actin was used as a loading control. Densitometry evaluation of each blot (normalized to actin) is shown on the right panel. Values were expressed as Mean ± S.E.M. (n = 4). Unpaired Student’s *t* test and one-way ANOVA were used to compare the means.

Given the extensive loss of numerous mitochondrial proteins related to mitochondrial dynamics during CS/Tx, it was important to show that not all mitochondrial proteins were decreased following CS/Tx. The major mitochondrial antioxidant protein, manganese superoxide dismutase (MnSOD, found in matrix) as well as Core2, a complex III subunit protein (found in inner membrane), were not reduced after CS alone or after CS/Tx (**Fig 7)**. These results indicate that CS/Tx does not appear to alter the expression of all mitochondrial proteins.

## Discussion

The current study clearly demonstrates for the first time that renal CS alone (18 hr) leads to damaged mitochondria and altered mitochondrial fission and fusion pathways. One important aspect of this study is the value of evaluating CS alone and combined with transplantation, since our data revealed complex I and II were partially inactivated with CS alone-*before transplantation*. Complex III activity was declined when rat kidneys exposed to CS were then transplanted (**Fig 2**), suggesting exacerbation of CS-induced mitochondrial dysfunction. The profound loss of ALL mitochondrial dynamic proteins examined (DRP1, MFF, OPA1, OMA1, YME1L, MFN1, MFN2) after CS/Tx may indicate altered mitochondrial biogenesis (synthesis), but the data showing no change in MnSOD or Core 2 indicate that this is unlikely. The reduced expression in the short form of OPA1 was unexpected and may indicate involvement of other protease systems (e.g. ubiquitin proteasome pathway) during CS/Tx, which deserves further study. Our recent study [23] using the renal autotransplantation (ATx) model showed no alteration in mitochondrial respiration. New studies shown here reveal that ATx lead to minimal effects on fission/fusion machinery, suggesting that CS plays an important role in disrupted mitochondrial function as well as fission and fusion.

OMA1 is considered a stress-induced protease that can function independently of ATP, whereas YME1L is constitutively active, requires ATP, and is needed for normal mitochondrial function [29;30;36;37]. As mentioned earlier, there are no assays available to measure OMA1 activity, which is certainly a drawback to studying this important mitochondrial protease. Many investigators have used OPA1 cleavage or altered OMA1 expression as indirect measures of OMA1 activity [30;37]. Consistent with this, our data, showing reduced expression of OMA1 and loss of the short form of OPA1, suggest OMA1 is activated during CS alone. Unexpectedly, after CS/TX we observed an abnormal expression/higher molecular weight species of OMA1 (**Fig 6**), suggesting possible binding to substrates or altered conformation of OMA1. To our knowledge very few studies have evaluated altered OMA1 protein levels in disease models, the majority of studies utilize labeled (e.g. myc tagged) overexpressed OMA1 in cell models exposed to different stressors. And since very little is understood about how OMA1 is regulated in disease, we postulate that our study will yield new information that will help unravel the complexities associated with this important mitochondrial protease.

In yeast it has been shown that OMA1 can exist as higher molecular weight oligomers in native gels, and that stress can alter OMA1 conformation and activity, but the mechanisms involved and whether this occurs in mammalian systems remains unknown [38;39]. Recently it was shown, in cell models, that OMA1 can degrade YME1L and vice versa [31], but very little is known about the inter-regulation of OMA1 and YME1L during disease. Dr. Luke Wiseman’s group has showed a reciprocal degradation of OMA1 and YME1L under different conditions using *in vitro* models [31]. These studies suggested that loss of YME1L actually serves to stabilize active OMA1 and can lead to mitochondrial damage. Therefore, given the extensive proteolysis of OPA1 and YME1L (a known substrate of OPA1) after CS/Tx we postulate that OMA1 is activated. It has been shown that loss of YME1L results in accumulation of non-assembled respiratory chain subunits [36], which may explain the enhanced damage to respiratory complex function observed after CS/Tx. In addition, YME1L normally degrades OMA1 to prevent excessive proteolytic processing in an ATP dependent manner [29;33]; however we recently showed that ATP is decreased following CS/Tx [23]. Thus, it is possible that CS/Tx leads to unregulated, overactive OMA1 which induces profound mitochondrial damage, but how and why this occurs is not known.

Very few disease models have demonstrated reduced expression of key mitochondrial fission and fusion proteins to the extent we have shown in the current study following CS/Tx. In most instances, enhanced fission (e.g. increased DRP1) and reduced fusion leads to the classic fragmented mitochondria along with a decline in mitochondrial function. For example, Funk and Schnellmann showed that expression of DRP1 increased and MFN2 declined after renal ischemia/reperfusion [40]. Another study showed that depletion of DRP1 leads to increased OPA1 proteolysis via OMA1 and that loss of DRP1 also leads to degradation of MFN1/2 via the ubiquitin proteasome system [41]. Interestingly, we also see a decline in DRP1 that precedes the reduced expression of MFNs, suggesting similar effects during CS/Tx. Since our study revealed decrease in all fusion/fission proteins examined after CS/Tx, further studies looking at different time points or with pharmacologic inhibitors of fusion or fission will be needed to determine precise cause/effect relationships. It is important to point out that dissection of such pathways in an animal model are much more difficult compared to cell or yeast models, but we believe assessment of mitochondrial dynamics in disease models are critically important.

There are a few limitations of our current study focused on selection of the animal model. Since the primary focus of this study was to evaluate the impact of CS alone, we chose to use a syngeneic (Lewis to Lewis) transplant model, rather than a more clinically relevant allograft (Fisher to Lewis) transplant model, which would otherwise add confounding effect from the immune system. Given our results showing profound renal and mitochondrial damage, we are concerned that adding an immune insult to the CS effect may increase mortality. Many studies using an allograft renal transplant model elect to keep the native kidney intact for up to ten days after surgery, presumably to prevent mortality. This paradigm would limit our ability to determine the acute (within 1–3 days) impact of CS plus immune activation on mitochondrial fission/fusion in the transplanted kidney. Another drawback to our model, is that we used static CS as the method of preservation, rather than the clinically preferred machine preservation, which involves constant pumping of cold preservation solution through the kidney. We selected the static CS method due to the obvious technical difficulty in pumping a rat kidney, but also because there are still many transplant centers that rely on static CS due to cost restrictions or to transportation of the donor kidney to the center. However, our prior publication using a novel method of cold machine preservation in a rat kidney *in vivo* revealed clear renal and mitochondrial damage [20].

**Fig 8:**
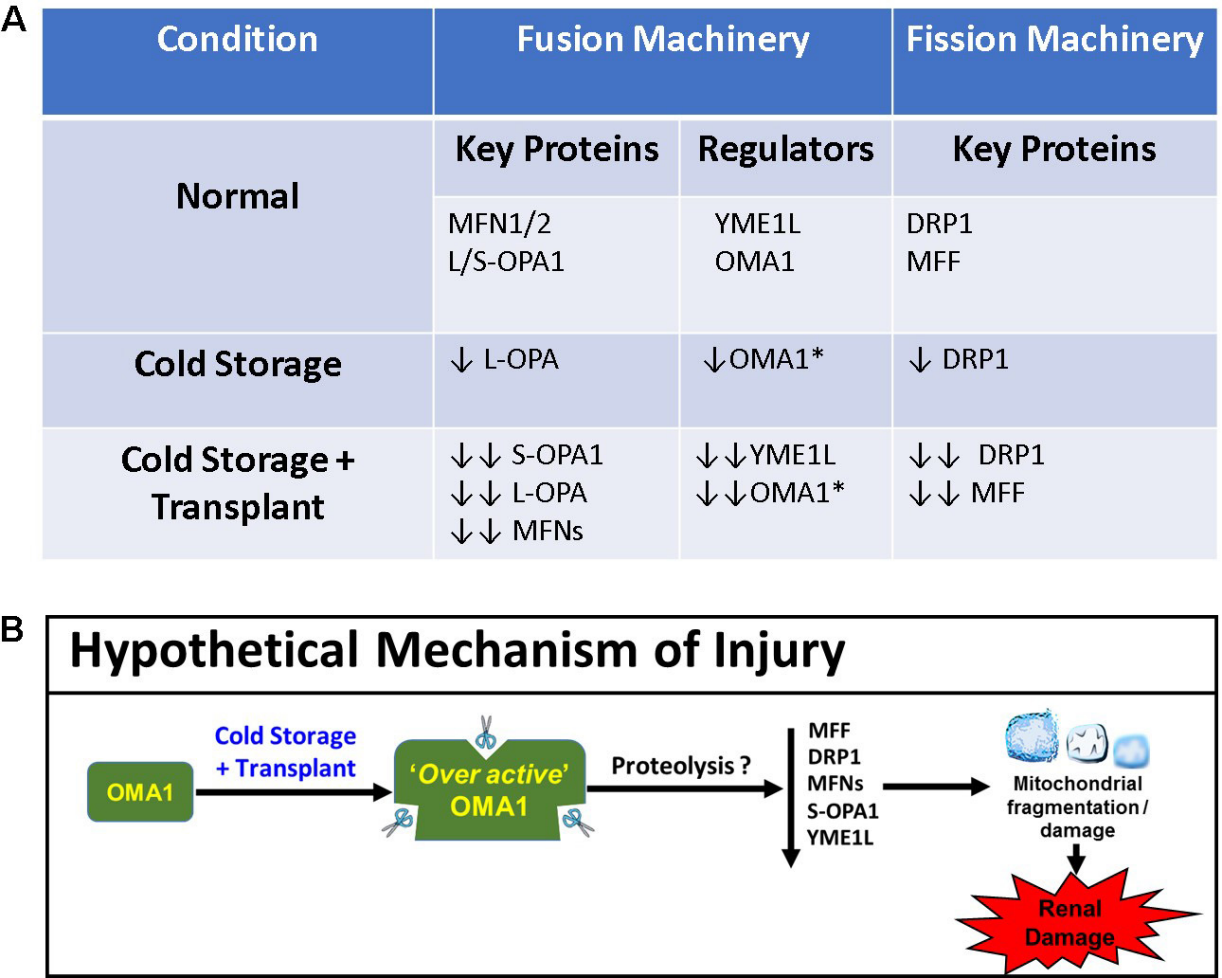
Summary of key findings and proposed hypothetical scheme. **A**. Summary table of key mitochondrial fission and fusion proteins affected by CS and CS/Tx. *Decrease in OMA1 expression equals increase in OMA1 activity [30;37]. **B.** Scheme representing a hypothetical mechanism of renal and mitochondrial injury during CS/Tx. Stress induced-mitochondrial protease OMA1 is over-activated when exposed to cold storage plus transplantation, which possibly contributes to proteolysis of critical fission and fusion proteins. This leads to fragmentation and damage of mitochondria and ultimately renal damage.

Long-term renal function after transplantation is improved when kidneys are harvested from living rather than deceased donors. The biggest difference in pre-surgical organ preparation is that deceased-donor kidneys are subjected to cold preservation (static or pumping) while a suitable recipient is located, whereas kidneys from living donors do not undergo lengthy cold preservation. The early work of Belzer and Southard revolutionized the world of preservation solutions [42–44] and made cold preservation of organs a clinical reality. The purpose of CS is to lower the kidney’s metabolic activity and oxygen demand during the wait for transplantation, but nevertheless, injury occurs. New methods need to be developed to blunt the extent of damage during renal CS, which would result in longer viable CS times, fewer discarded kidneys, more transplants, and better function post-transplantation. Our prior studies showed that CS induces ROS and that CS injury was protected by using a mitochondrial-targeted antioxidant, MitoQ [19;22]. Interestingly, a recent paper by Rainbolt et al. showed that cells treated with hydrogen peroxide lead to OMA1-dependent OPA1 processing [33]. We hypothesize that CS combined with transplantation induces OMA1 (possibly via increased ROS) to become ‘over active’ via a possible altered conformation (e.g. post-translational modifications or altered binding to substrates), leading to degradation of YME1L, further loss of DRP1 (which may contribute to degradation of MFNs [41]), and profound mitochondrial and renal damage (**Fig 8**). Pharmacological strategies designed to blunt the OMA1 mediated effect could prove to be translationally beneficial.

## Supporting information

S1 File ARRIVE checklist. (PDF)
